# Efficient assembly and annotation of the transcriptome of catfish by RNA-Seq analysis of a doubled haploid homozygote

**DOI:** 10.1186/1471-2164-13-595

**Published:** 2012-11-05

**Authors:** Shikai Liu, Yu Zhang, Zunchun Zhou, Geoff Waldbieser, Fanyue Sun, Jianguo Lu, Jiaren Zhang, Yanliang Jiang, Hao Zhang, Xiuli Wang, KV Rajendran, Lester Khoo, Huseyin Kucuktas, Eric Peatman, Zhanjiang Liu

**Affiliations:** 1 The Fish Molecular Genetics and Biotechnology Laboratory, Department of Fisheries and Allied Aquacultures and Program of Cell and Molecular Biosciences, Aquatic Genomics Unit, Auburn University, Auburn, AL, 36849, USA; 2 The Shellfish Genetics and Breeding Laboratory, Fisheries College, Ocean University of China, Qingdao, 266003, P.R. China; 3 USDA, ARS, Catfish Genetics Research Unit, 141 Experiment Station Road, Stoneville, Mississippi, 38776, USA; 4 College of Veterinary Medicine, Mississippi State University, 127 Experiment Station Road, Stoneville, Mississippi, 38776, USA

## Abstract

**Background:**

Upon the completion of whole genome sequencing, thorough genome annotation that associates genome sequences with biological meanings is essential. Genome annotation depends on the availability of transcript information as well as orthology information. In teleost fish, genome annotation is seriously hindered by genome duplication. Because of gene duplications, one cannot establish orthologies simply by homology comparisons. Rather intense phylogenetic analysis or structural analysis of orthologies is required for the identification of genes. To conduct phylogenetic analysis and orthology analysis, full-length transcripts are essential. Generation of large numbers of full-length transcripts using traditional transcript sequencing is very difficult and extremely costly.

**Results:**

In this work, we took advantage of a doubled haploid catfish, which has two sets of identical chromosomes and in theory there should be no allelic variations. As such, transcript sequences generated from next-generation sequencing can be favorably assembled into full-length transcripts. Deep sequencing of the doubled haploid channel catfish transcriptome was performed using Illumina HiSeq 2000 platform, yielding over 300 million high-quality trimmed reads totaling 27 Gbp. Assembly of these reads generated 370,798 non-redundant transcript-derived contigs. Functional annotation of the assembly allowed identification of 25,144 unique protein-encoding genes. A total of 2,659 unique genes were identified as putative duplicated genes in the catfish genome because the assembly of the corresponding transcripts harbored PSVs or MSVs (in the form of pseudo-SNPs in the assembly). Of the 25,144 contigs with unique protein hits, around 20,000 contigs matched 50% length of reference proteins, and over 14,000 transcripts were identified as full-length with complete open reading frames. The characterization of consensus sequences surrounding start codon and the stop codon confirmed the correct assembly of the full-length transcripts.

**Conclusions:**

The large set of transcripts assembled in this study is the most comprehensive set of genome resources ever developed from catfish, which will provide the much needed resources for functional genome research in catfish, serving as a reference transcriptome for genome annotation, analysis of gene duplication, gene family structures, and digital gene expression analysis. The putative set of duplicated genes provide a starting point for genome scale analysis of gene duplication in the catfish genome, and should be a valuable resource for comparative genome analysis, genome evolution, and genome function studies.

## Background

Recent advances in next-generation sequencing enabled an array of whole genome sequencing or re-sequencing projects in both model and non-model species. Such efforts have produced a wealth of genome resources. However, thorough genome analysis thereafter is essential to associate genome sequences with biological meanings [1,2]. An important step in genome analysis is to decipher the complete protein coding sequence (CDS) region of each gene. In eukaryotes, prediction of CDS regions in genomic sequence is complicated by the intron interruptions and the low proportion of protein coding regions in the genome. It is still problematic at present to predict the correct distribution of CDS regions solely based on genomic sequences [3]. To obtain information about the portion of a genome that is transcribed as RNAs and then translated into proteins, a comprehensive set of full-length transcripts is needed [4].

In teleost fish, genome annotation is further seriously hindered by genome duplication. Because of gene duplications, it’s unable to establish orthologies simply by homology comparisons. Rather intense phylogenetic analyses or syntenic analyses of orthologies are required for the identification of genes. To conduct phylogenetic analysis and orthology analysis, full-length coding regions of transcripts are essential. Furthermore, the nature of highly diversified and duplicated genome of fish species hindered sequence assembly and complicated the genome annotation as well as SNP identification. Previous efforts aiming at SNP discovery for catfish rendered the issue about discrimination of false positive SNPs derived from paralogous sequences and multisite sequences (PSVs/MSVs) [5].

Obtaining large numbers of full-length transcripts is not an easy task. In most cases, full-length transcripts were obtained by Sanger-based sequencing of full-length cDNA clones. This strategy works well for many highly abundantly expressed and short transcripts because in such cases: 1) clones containing full-length transcripts can be readily identified, and 2) complete sequencing can be achieved through sequencing from both ends of the clones. However, such a task for rarely expressed genes and large transcripts can be troublesome because the identification of full-length cDNA containing clones is itself a huge challenge, and even if the clones are identified, complete sequencing of long inserts using Sanger sequencing can still be time-consuming and expensive. Recently, next-generation sequencing has been recognized as one solution for transcriptome sequencing at a reasonable cost [6,7]. High-throughput sequencing of cDNA (RNA-Seq) does not rely on prior knowledge, enabling interrogation of all transcripts including potentially novel transcripts uncaptured in Sanger-based sequencing [8]. RNA-Seq can provide sufficient sequencing coverage on whole transcriptome scale to ensure the precision of each single base and integrality of full-length transcripts [7,9]. However, huge amount of short reads generated from RNA-Seq make the transcriptome assembly difficult, which is not only impeded by repeats but also by alternatively spliced transcripts. Moreover, while genomic sequencing coverage is generally uniform across the genome, transcriptome sequencing coverage is highly variable, depending on gene expression levels, excluding the use of coverage information to resolve repeated motifs [10]. Since a transcriptome assembly with good quality is essential for all the downstream analysis, extra efforts are required to improve the transcriptome assembly. An optimized assembly strategy can be obtained by combinatory use of different assembly softwares, especially the ones using multiple *k*-mers to resolve the problem of biased coverage of transcriptome sequencing. A higher *k*-mer length will theoretically results in a more contiguous assembly of highly expressed transcripts. On the other hand, poorly expressed transcripts will be better assembled if lower *k*-mer lengths are used [10]. Therefore, multiple *k*-mer approach can be used to increase the assembly sensitivity and contiguity. With the fast development of assemblers able to efficiently handle a greater number of sequence reads [10,11], short-reads can be of considerable utility for assembling transcriptomes of non-model organisms [9,12,13]. The most significant issue with RNA-Seq for the assembly of transcriptome, however, is the allelic variation. Most vertebrate species are diploid organisms and therefore two sets of chromosomes are involved in the generation of transcripts. Even if only one individual is used for RNA-Seq, the assembly of related transcripts from the two sets of chromosomes creates “haplotypes” that do not exist, and this alone prohibits assembly of full-length transcripts from multiple sequences. To overcome this huge problem, the ideal approach is to create an individual with doubled haploid genome and therefore there are no allelic variations such that the short reads from RNA-Seq can be assembled, not only technically feasible by the software packages, but also biologically meaningful as they are transcribed from the same sequences.

In catfish, years of efforts have resulted in nearly 500,000 quality ESTs [14], but a limited number of full-length transcripts were obtained through the traditional clone-by-clone approach using Sanger sequencing [15]. Recent efforts using RNA-Seq allowed the identification of a large number of transcripts in catfish [4], but the assembly of full-length transcripts was hindered by the reasons discussed above. In order to circumvent this problem and generate a large set of full-length coding sequences to support the genome sequencing and annotation in catfish, doubled haploid catfish was produced that has been demonstrated to harbor two sets of homozygous chromosomes [16]. Such homozygous catfish is ideal material for the generation and assembly of full-length transcripts using RNA-Seq. Here we report deep sequencing of catfish transcriptome by RNA-Seq using this gynogenetic homozygous catfish. The two main objectives of this study were to develop a comprehensive set of reference transcript sequences for genome-scale gene discovery and expression studies in catfish; and to obtain a large number of full-length transcripts for whole genome annotation, duplicate gene identification, and facilitating detection of false SNPs derived from PSVs/MSVs.

## Results

### Transcriptome sequencing

Illumina sequencing was conducted to generate short reads of transcripts from a doubled haploid channel catfish. The cDNA was made from pooled RNA samples isolated from 19 tissues of the catfish (See Methods for details). High throughput sequencing was conducted using the Illumina Hiseq 2000 platform to generate 100-bp paired-end reads. A total of 315,703,698 reads (157,851,849 from each end) were generated. After removal of ambiguous nucleotides, low-quality reads (quality scores < 20) and sequences less than 15 bp, over 300 million reads totaling 27.1 billion bases were obtained for further analysis (Table 1).

**Table 1:** Summary of data generated for catfish transcriptome using Illumina Hiseq 2000 sequencing

**Sequencing**	No. of tissues*	**No. of raw reads**	**Read length (bp)**	**No. of reads after trim**	**Avg. length after trim (bp)**	**No. of bases after trim (Gbp)**
s_4_1_1	19	49,077,054	100	48,743,295	92.8	4.5
s_4_1	19	128,862,236	100	124,882,873	87.6	10.9
s_5_1	19	137,764,408	100	133,926,741	87.1	11.7
Total	-	315,703,698	100	307,552,909	88.0	27.1

*Sequencing was conducted using the total RNA isolated from nineteen tissues of a doubled haploid channel catfish. Tissues include head kidney, fin, pancreas, spleen, gill, brain, trunk kidney, adipose, liver, stomach, gall bladder, ovary, intestine, thymus, skin, eye, swim bladder, muscle, and heart.

### Transcriptome assembly

In order to obtain a comprehensive and reliable assembly, three different assemblers were used including CLC Genomics Workbench (version 4.2), ABySS (version 1.2.6) and Velvet (version 1.1.02) for *de novo* assembly. Although all these three assemblers use the same de Bruijn graph algorithm, they are different in how to treat sequencing errors, resolve ambiguities and utilize read pair information [17]. Furthermore, multiple *k*-mer approach applied in ABySS and Velvet has the capability of producing a better assembly for transcripts from both highly and lowly expressed genes. Therefore, we believe that the combinatory use of these three assemblers could improve the assembly.

As shown in Table 2, the ABySS assembly generated 192,558 contigs with minimum length of 200 bp, and generated the highest N50 and average contig lengths (1,888 bp and 1,004 bp, respectively). The CLC assembly resulted in a total of 217,114 contigs (≥ 200 bp) with N50 length of 1,120 bp, and average contig length of 658 bp. The contigs from Velvet assembly were relatively short with N50 and average contig lengths of 809 bp and 580 bp, respectively. Though a smaller number of contigs with minimum length of 200 bp were assembled in the ABySS assembly, it allowed the assembly of the largest number of contigs with length greater than 1 kb among the three assemblers (56,229 contigs ≥1 kb).

**Table 2:** **Summary of assemblies generated using various *de novo *assemblers**

Assemblies*	**No. of contigs ≥200bp**	**No. of contigs with length ≥N50**	**No. of contigs with length ≥1kb**	**Avg. contig length (bp)**	**N50 (bp)**	**Total size (Mbp)**
CLC	217,114	29,564	33,332	658	1,120	142.8
ABySS	192,558	28,791	56,229	1,004	1,888	193.5
Velvet	311,734	56,471	42,685	580	809	181.0
Final merged	370,798	48,730	68,569	743	1,395	275.5

*CLC denotes the assembly generated by CLC Genomics Workbench, ABySS denotes the assembly generated by ABySS with multiple *k*-mers and merged by running the first stage of Trans-ABySS pipeline, Velvet denotes the assembly generated by Velvet with multiple *k*-mers and merged by running the first stage of Trans-ABySS pipeline, and Final merged denotes the final assembly generated by combining the three sets of assembly and retain only one of longest contigs for the ones assembled by more than one assemblers.

The multiple *k*-mer assemblies encompass both higher *k*-mer lengths to result in a more contiguous assembly of highly expressed transcripts, and lower *k-*mer lengths to better assemble poorly expressed transcripts to increase assembly sensitivity. In order to compare the ability of each assembler to reconstruct protein-coding gene transcripts on sensitivity and contiguity, each assembly was searched against annotated protein database using BLASTX (Additional file 1). The significant unique protein hits were identified from both zebrafish RefSeq protein and Uniprot/Swiss-Prot database with E-value cutoff of 1e-10. The largest number of unique protein hits was obtained by Velvet assembly in comparison to the assemblies generated by CLC and ABySS. The contigs generated by ABySS showed higher N50 and average lengths, but a smaller number of unique protein hits was obtained (Table 2; Additional file 1). By comparison, the contigs generated from CLC offered larger number of unique protein hits than ABySS, while providing higher N50 than Velvet.

A total of 370,798 non-redundant transcript-derived contigs were obtained as the final assembly based on the three sets of assemblies. The combined final assembly had a N50 length of 1,395 bp and average contig length of 743 bp, including 68,569 contigs with length greater than 1 kb (Table 2). Such assembly took advantage of each assembler to achieve both highest assembly sensitivity and contiguity. In order to assess the transcriptome capture obtained by the current work, we aligned all the channel catfish ESTs currently available in NCBI (354,488) with transcripts of the final assembly reconstructed in this study. The results showed that the vast majority (97% of the total) of the ESTs were represented in our data set showing ≥ 90% identity over a length of ≥ 100 bp and E-value of ≤ 1e-10. All the 370,798 transcript-derived contigs have been submitted to the NCBI Transcriptome Shotgun Assembly (TSA) database under accession numbers: JT123347-JT494144.

### Assessment of transcriptome assembly

We previously generated a total of 1,087 full-length cDNAs from channel catfish by direct sequencing of full-length cDNA clones [15]. This independently generated full-length cDNAs provided the material basis for the evaluation of the transcriptome assembly presented here, at least partially. The previously sequenced full-length cDNA sequences were used as queries to compare the transcript contigs assembled in this study. Based on the comparison, a general assessment can be obtained on what proportion of transcripts was successfully reconstructed with full-length in this work. The results were summarized in Table 3. Of the 1,087 full-length cDNAs, 1,011 (93%) were reconstructed completely with the same open reading frames (ORFs) in the present study. Among the remaining 76 transcripts, 45 transcripts were almost fully assembled, but lacking some sequences at either 5’ end or 3’ end, and 31 transcripts (< 3%) were poorly assembled in the current study which did not get any specific match with contigs in the final assembly (Table 3). These results demonstrated that the assembly quality was highly reliable; and a small fraction of transcripts that cannot be assembled were due to the absence of sequences in the current RNA-Seq sequence pool.

**Table 3:** Assessment of transcriptome assembly

	**Number**	**Percentage**
Total previous full-length cDNAs	1,087	
Completely assembled with same ORFs	1,011	93.0%
Partially assembled	45	4.1%
Poorly assembled	31	2.9%

A total of 1,087 previously identified catfish full-length cDNAs were used to compare with the assembled transcripts in this study to assess the transcriptome assembly.

### BLASTX annotation based on homology search

To identify the putative function of catfish transcripts, all the sequences were blasted against the reference proteins available in NCBI RefSeq and Uniprot/Swiss-Prot protein databases using BLASTX with E-value ≤ 1e-10. A total of 87,931 (23.7%) catfish contigs had significant hits to zebrafish RefSeq proteins corresponding to 19,711 unique proteins. When blasted against the Uniprot database, 80,012 (21.6%) catfish contigs had significant hits, corresponding to a total of 26,396 unique accession numbers and 17,669 unique proteins. Cumulatively, a total of 94,476 (25.5%) catfish contigs had significant hits to known proteins from either NCBI RefSeq or Uniprot, corresponding to a total of 25,144 unique proteins (Table 4). The remaining contigs may represent UTRs, non-protein coding genes or additional transcripts from catfish-specific genes which are too divergent to be annotated by homology search with current E-value cutoff.

**Table 4:** Summary of BLASTX searches to annotated protein databases for final assembly

	**No. of contigs with hits**	**No. of unique accessions**	**No. of unique protein hits**
Zebrafish RefSeq	87,931	19,711	19,711
Uniprot/Swiss-Prot	80,012	26,396	17,669
Total	94,476	-	25,144

Final assembly containing 370,798 contigs was used to search the zebrafish RefSeq protein database and the Uniprot database to identify the homologous genes represented by the catfish expressed sequences.

### Analysis of BLAST top hit species and potential presence of non-catfish transcripts

**Figure 1:**
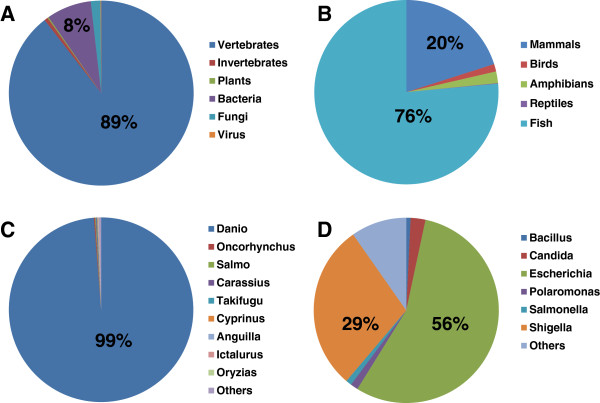
**Distribution of taxonomic groups of BLAST top hit species. **(**A**) The top hit species of BLAST searches are categorized into vertebrates, invertebrates, plants, bacteria, fungi and virus, and their percentages are presented. (**B**) The top hit species of BLAST searches within vertebrates are sub-categorized into mammals, birds, amphibians, reptiles, and fish, and their percentages are presented. (**C**) The top hit species of BLAST searches within fish are sub-categorized into various fish species as indicated, and their percentages are presented. (**D**) The top hit species of BLAST searches within bacteria are sub-categorized into various bacterial species as indicated, and their percentages are presented.

In addition to its high capability of capturing transcripts from the target species, high throughput transcriptome sequencing using the next-generation technology had enabled capture of expressed sequences from xenobiotic species that are commensal with the species of study [18,19]. In this study, total RNA of catfish samples was isolated from 19 tissues including intestine and stomach that are particularly prone to contamination of xenobiotic species. Therefore, it is possible that some of the sequences in our dataset are from xenobiotic species that are commensal with catfish. We, therefore, conducted BLASTX analysis and attempted to determine species with which the top hits were generated. Our data showed that 89% of our sequences with a significant BLAST hit had the top hits from a vertebrate species (Figure 1A), and of these 76% had top hits from fish sequences (Figure 1B). Among the sequences with top hits from fish, 99% had top hits with zebrafish, as expected with the phylogenetic relationship (Figure 1C).

However, it’s noteworthy to mention that a good fraction (1,972 sequences, 8%) of our sequences had their top BLASTX hits with bacterial sequences, followed by those had top hits with fungi (424 sequences, 2%), and a few (33 sequences) even had top hits to viral sequences. Of these 2,429 sequences with top hits to other species than vertebrates, the top BLAST hit e-values were larger than those with top hits with vertebrates, in the range of 10^-10^ to 10^-40^, whereas those with top hits to vertebrate species had a smaller e-value (often < 10^-80^), suggesting the presence of xenobiotic species in the catfish samples. The top BLAST hits to bacteria were from a variety of species, of which 56% were *Escherichia coli*, and 29% were *Shigella* species (Figure 1D). Since our sequences were derived from multiple tissues, some of the bacterial sequences could belong to commensal microorganisms in digestive organs, although careful treatments had been taken to clean the tissues during collection process. Further investigation is warranted to fully understand these sequences most similar to xenobiotic species, which could provide information concerning symbionts and other microbial communities in catfish.

### Conserved domain (CD) search for contigs without BLAST protein hits

**Figure 2:**
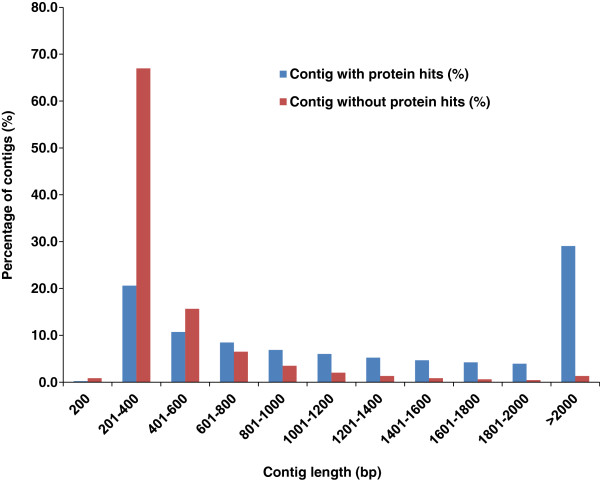
**The contig length comparison between contigs with and without protein hits.** The X-axis represents the contig length, and Y-axis represents the percentage of contigs. Note the high percentage of contigs that do not have significant protein hits in public protein database are in short length (83% less than 600 bp), and the high proportion of contigs with protein hits are long contigs.

After the annotation for catfish contigs based on homology search using BLASTX, a total of 276,322 catfish contigs did not get any significant protein hits from either zebrafish RefSeq or Uniprot protein database. It is reasonable to think that some protein-coding gene-derived contigs fail to get significant protein hits due to their short lengths. A comparison between the contigs with and without significant protein hits was conducted as shown in Figure 2. The majority of contigs that do not have significant protein hits from public protein database are with short length (83% less than 600 bp), while over 50% of contigs with protein hits are with length greater than 1 kb.

In order to identify potential protein-coding genes from those contigs without significant blast hits from protein databases, we conducted *ab initio* prediction of the potential ORFs for the 276,322 catfish contigs that did not have significant hits to known proteins. A total of 260,793 contigs have putative ORFs detected with minimum length of 30 amino acids, and 16,688 of which possess putative ORFs with minimum length of 100 amino acids (Additional file 2). To determine the putative functions of these ORFs, the CD-Search tool in NCBI was used to identify conserved domains, or functional units, within the protein query sequences. The specific hit found by a CD-Search indicates a high confident association between the protein query sequence and a conserved domain, resulting in a high confidence level for the inferred function of the protein query sequence. A total of 4,984 ORFs were identified with conserved domains (Additional file 3), suggesting that such ORF-harboring contigs were derived from functional protein-coding genes as well.

### Comparison of catfish transcripts with model fish species

**Figure 3:**
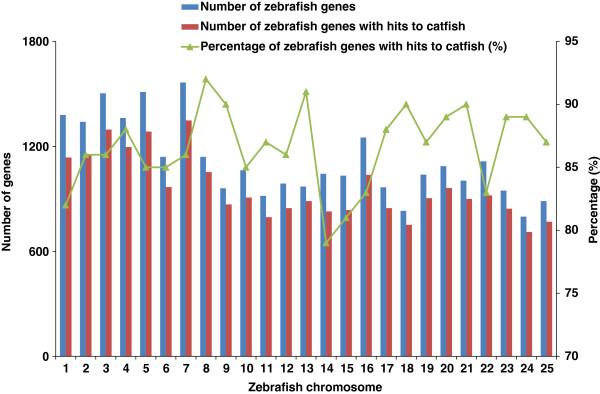
**Homologous distribution of identified catfish genes on zebrafish chromosomes.** X-axis represents 25 zebrafish chromosomes. The left Y-axis represents the number of genes, and right Y-axis is the percentage of zebrafish genes on each chromosome identified in catfish.

In order to assess the capture of transcriptome obtained by catfish, reciprocal comparisons were conducted with the five model fish species with sequenced genome in Ensembl database including zebrafish, fugu, medaka, stickleback and *Tetraodon*. First, the protein sequences of the five sequenced fish species were mapped to the catfish transcripts using TBLASTN. From this, 39,250 (96.7%) of the 40,585 zebrafish proteins were matched, corresponding to 25,330 (96.9%) of the 26,152 Ensembl genes. The percentages of protein and gene match in other four fish were comparable to that observed in zebrafish, suggesting a high degree of transcriptome coverage obtained in catfish. Second, the catfish transcripts were mapped to the proteins of the five sequenced fish species using BLASTX (E-value ≤ 1e-10) to estimate the number of transcripts and genes represented in catfish. Based on zebrafish dataset, a total of 24,281 Ensembl zebrafish proteins were matched by catfish contigs, corresponding to 20,014 unique genes (Table 5). As indicated in Figure 3, zebrafish genes that had significant hits to catfish were relatively evenly distributed across 25 chromosomes (with the percentages ranging from 80% to 92%), suggesting the comprehensive capture of transcriptome for catfish genes on the genomic scale.

**Table 5:** Reciprocal BLAST comparison between catfish and five model fish with sequenced genome

**Species**	**#Total proteins**	**#Protein coding genes**	**#Proteins have hits in catfish**	**#Genes have hits in catfish**	**#Unique protein hits by catfish**	**#Unique gene hits by catfish**
zebrafish	40,585	26,152	39,250	25,330	24,281	20,014
fugu	47,841	18,523	47,583	18,336	26,230	15,834
medaka	24,661	19,686	23,311	18,465	18,200	16,007
stickleback	27,576	20,787	26,375	19,740	19,503	16,835
*Tetraodon*	23,118	19,602	22,648	19,208	17,636	15,996

First, protein sequences of five model fish in Ensembl database were searched against catfish contigs using TBLASTN to assess the extent of reference sequences that were observed in catfish; reciprocally, all catfish contigs were searched against protein sequences of five model fish using BLASTX to assess the number of genes represented by the catfish sequences when compared to those in each of the five species.

### Identification of gene duplicates

**Figure 4:**
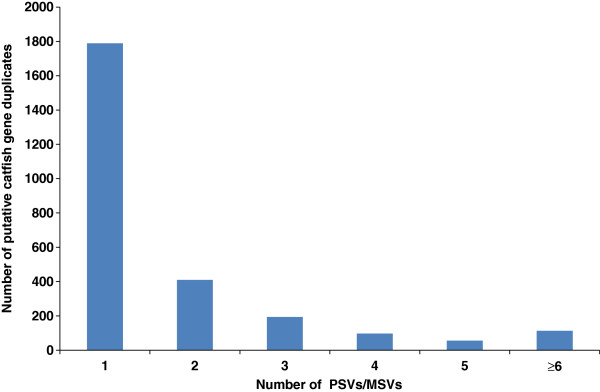
**Detection of putative catfish gene duplicates.** X-axis represents the number of PSVs or MSVs detected, while the Y-axis is the number of putative duplicated genes in catfish that contained the PSVs or MSVs.

The highly diversified and duplicated genome hindered sequence assembly and complicated the genome annotation as well as SNP identification. Duplicated regions contain paralogous sequence variants (PSVs) or multisite variants (MSVs) which are readily mistaken for SNPs [20,21]. In this work, a doubled haploid channel catfish individual was used, which provided the opportunity to examine the gene duplication in catfish at genome-scale. Such analysis was based on a simple principle that the doubled haploid channel catfish, which had two sets of identical chromosomes, should not contain allelic variations. Therefore, the transcripts should be derived from duplicated gene copies once there was any ‘SNP’ detected (Additional file 4). All the 25,144 contigs with unique protein hits were used for detection of “SNPs” as in previous study [5]. In this analysis, a total of 4,878 PSVs or MSVs were detected from 2,692 transcripts accounting for 2,659 unique genes. Among the genes with PSVs or MSVs detected, the majority (67.3%, 1,789/2,659) contained only one PSV or MSV, while 113 (4.2%) genes contained greater than five PSVs or MSVs (Figure 4). These genes are putative gene duplicates in the catfish genome.

To evaluate the detected gene duplicates, we aligned the transcripts to the preliminary assembly of the catfish genome (255,858 contigs with mean length of 2,996 bp and N50 of 6,027 bp, unpublished data). Duplicated genes were expected to be present in different genome locations (generally, i.e., genome contigs), and thus on different genomic contigs. As summarized in Additional file 5, the vast majority (92%, 2,446/2,659) of transcript-derived contigs hit more than one genomic contigs, suggesting their potential involvement in duplication. Of the remaining 213 transcript contigs, 196 contigs had only one catfish genome contig hits, indicating their uniqueness in the catfish genome. However, the possibility that it was because of the lack of corresponding genome contigs in the preliminary catfish genome assembly cannot be excluded. A total of 17 gene-derived transcripts did not get any significant match with genome contigs, suggesting the incompleteness of the current catfish preliminary assembly (Additional file 5). Additional analysis of paralogous relationships may be strengthened by examination of physical locations in the genome for tandom duplications and flanking regions for segmental duplications when catfish genome scaffolds become available in the near future. It is understood that the same gene can still be placed into two or more genomic contigs with the preliminary assembly, but this problem should be overcome soon when the catfish genome assembly is completed.

### Identification of catfish full-length transcripts

It has been shown that transcriptome sequencing using RNA-Seq can be a cost-effective approach for reconstruction of full-length transcripts in species without a reference genome [9]. In the context of this work, we took advantage of an individual homozygous catfish which provided a haploid transcriptome to facilitate the transcriptome assembly. A large fraction of transcripts were reconstructed and a large portion of these reconstructed transcripts were expected to be full-length transcripts. To identify full-length transcripts in our transcript collection, we utilized the functional annotation results with all the contigs in the final assembly being searched against zebrafish RefSeq and Uniprot database. The ORFs were predicted by using BLASTX-aided method which detects ORFs by finding the starting methionine and stop codon in catfish transcripts relative to the same features in the most closely related reference proteins identified by BLASTX.

**Figure 5:**
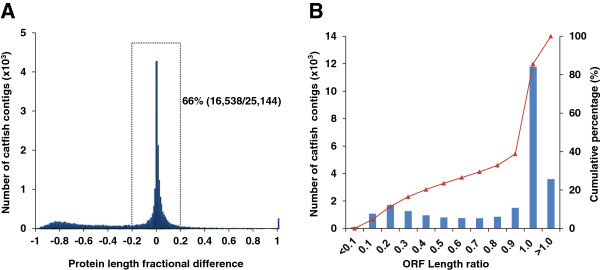
**Comparison of the lengths of deduced catfish proteins with homologous reference proteins from databases.** (**A**) Fractional distribution of catfish proteins with lengths falling within various fractions, e.g., 0 indicates identical lengths of catfish proteins with reference proteins; -0.2 and 0.2 indicates the lengths of catfish proteins are 20% shorter or longer than those of reference proteins, respectively, and so on. Overall 66% or 16,538 transcripts out of a total of 25,144 identified unique catfish genes are within 80% bracket as compared with the lengths of reference protein counterparts. (**B**) Ratio of catfish predicted protein length versus length of reference protein was indicated in histograms (left Y axis), and the curved line denotes the cumulative percentage (right Y-axis). X-axis is the ratio of predicted catfish protein length to corresponding reference orthologous protein length, i.e. catfish protein length/reference protein length. Note that the unit of X-axis is ten times of that in (**A**), i.e. the ratio of 1.0 represents the transcripts of length within 95% bracket as compared with the lengths of reference protein counterparts, etc. The left Y-axis represents the number of occurrence of catfish protein lengths in thousand, and right Y-axis is the cumulative percentage.

The lengths of protein sequence, translated from the catfish transcripts which had significant protein hits from protein database, ranged from 21 to 12,863 amino acids (aa). The relationship between catfish proteins and homologous reference proteins is shown in Figure 5. As shown in Figure 5A, the most common occurrence is when the catfish protein and reference protein lengths are identical, which occurs with 17% (4,282/25,144) of the catfish transcripts with unique protein hits. The majority of catfish transcripts (66%) have translated proteins within ± 20% of their reference protein lengths (Figure 5A).

In the present study, we defined the full-length transcript as a consensus sequence containing the complete CDS including the translational start codon ATG and the termination codon TAA, TAG, or TGA. To get an estimate of the number of transcripts containing full-length coding sequences, we used the ORF length ratio (catfish predicted protein length/reference protein length) to assess the completeness of CDS in each transcript. As indicated in Figure 5B, over 60% of catfish contigs with unique protein hits contain predicted CDS with comparable length to reference protein (ORF ratio ≥ 1.0, 15,397/25,144). A total of 16,869 contigs with ORF ratio ≥ 0.9 potentially harboring full-length coding sequences were selected for further manual inspection (Table 6).

**Table 6:** Summary of full-length transcript identification after manual inspection

	**Number**
Potential full-length transcripts	16,869
Full-length transcripts	14,240
Transcripts lack at 5' end	1,725
Transcripts lack at 3' end	711
Transcripts with incorrect ORFs	193

A total of 16,869 potential full-length transcripts that matched 90% length of reference proteins were selected for manual inspection. Full-length transcripts were defined as transcripts contain a complete CDS if the ORF revealed a start codon and stop codon in agreement with the match in the database.

Manual inspection of completeness of CDS in each transcript sequence was determined by the BLASTX alignment. In this procedure, we consider a full-length transcript to contain a complete CDS if the ORF revealed a start codon and stop codon in agreement with the matched protein sequence in the database. As summarized in Table 6, a total of 14,240 were identified as full-length transcripts with complete coding sequences, 1,725 contigs and 711 contigs were partial transcripts lacking of 5’ end or 3’ end, respectively, and 193 contigs had incorrect open reading frames predicted due to either partial sequences or incorrect bases in start codon regions.

**Figure 6:**
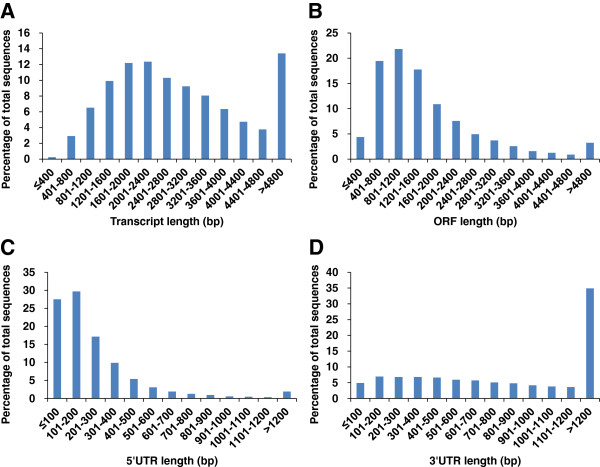
Length distributions of putative catfish full-length transcripts (A), ORF (B), 5’-UTR (C), and 3’-UTR (D).

As shown in Figure 6, the majority of full-length transcripts were with lengths ranging from 1 kb to 4 kb with the average of 3,006 bp. The lengths of ORFs ranged from 132 bp to 15,684 bp, with an average length of 1,654 bp. The lengths of 5’ UTRs were relatively short, with the average of 254 bp, while the 3’ UTRs were longer with the average length of 1,096 bp. It is noteworthy that a large proportion of full-length transcripts contained “very” long 3’ UTRs (35% transcripts with length greater than 1.2 kb). This is quite different from the results obtained in our previous work where most of the 3’ UTRs had lengths less than 400 bp [15]. As indicated before, the bias of cDNA library creation and the selection process towards smaller transcripts could be the major reason for the short 3’ UTRs in previous study. The results shown here indicated that RNA-Seq for the full-length transcripts used in present work was capable to reconstruct most of the transcripts including the ones with long ORFs and 3’ UTRs that are difficult for full-length cDNA clone sequencing to get the full-length.

**Figure 7:**
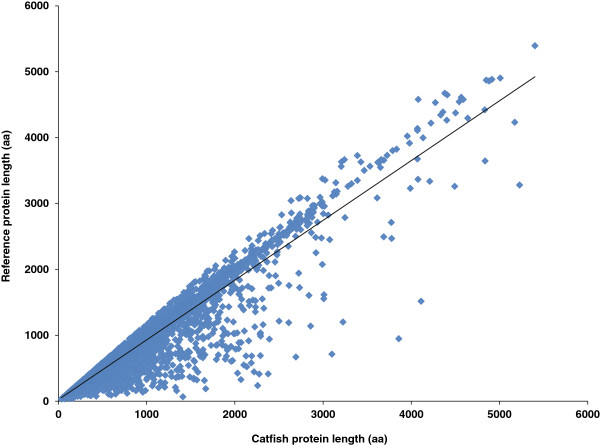
**Length comparisons of deduced catfish ORFs of the assembled full-length transcripts with homologous reference proteins.** X-axis: catfish predicted protein length (amino acids), and Y-axis: reference protein length (amino acids).

The protein sequences translated from catfish full-length transcripts had lengths ranging from 44 to 5,228 amino acids (aa). The majority of catfish proteins lengths were similar to that of their homologs. The relationship between catfish proteins and reference proteins from related species is illustrated in Figure 7, where the catfish protein lengths are plotted against the corresponding reference protein lengths. The majority of catfish proteins had same lengths as their homologous proteins in other species, but some are longer while others are shorter (Figure 7).

### Examination of sequences surrounding the start codon and stop codon of the catfish full-length transcripts

**Figure 8:**
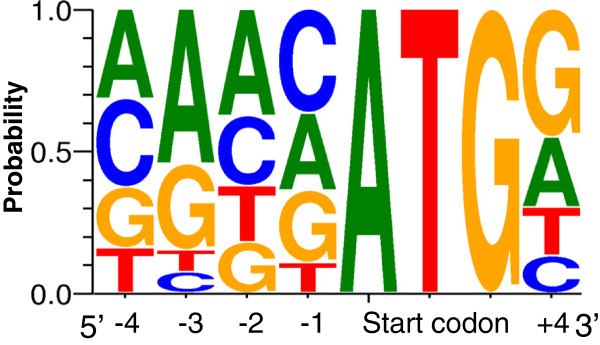
**Analysis of Kozak consensus sequences surrounding the start codon AUG in the catfish full-length transcripts.** Kozak consensus sequences were illustrated by WebLogo using stacks of symbols, one stack for each position in the sequence. The size of symbols within the stack indicates the relative frequency of each base at that position.

The AUG start codon context, also referred to as the Kozak motif, was reported as a consensus sequence for initiation of translation in vertebrates [22]. The catfish Kozak motif, spanning the position −4 to +4 was illustrated using WebLogo [23] as shown in Figure 8. The bases most frequently observed in the catfish Kozak motif are AAACATGG with the start ATG codon underlined, which is same as the results obtained in our previous work [15]. The most conserved bases are, as in other species, position −3 (A/G), the start codon (ATG) and position +4 (G). The consensus sequence of Kozak motif is reported as CACCATGG in mammals [24], with the start ATG codon underlined. The most frequently observed bases in the Kozak motif of Atlantic salmon, *Salmo salar*, were CAACATGG [25]. Catfish Kozak consensus sequence appears to be highly similar to *S. salar* except that an adenine base instead of cytosine base was observed as the −4 position. The conservation of the catfish Kozak consensus sequence, as with the 5’ UTR analysis, provided additional support for the proper identification of the start codon ATG in this work.

**Figure 9:**
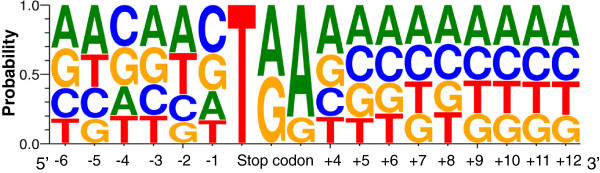
**Analysis of consensus sequences surrounding the stop codon in catfish full-length transcripts.** The consensus sequences were illustrated by WebLogo using stacks of symbols, one stack for each position in the sequence. The size of the symbols within the stack indicates the relative frequency of each base at that position.

The translation termination was a very complex process, which involved stop codon recognition, peptidyl-tRNA hydrolysis and release of ribosome from the mRNA [26,27]. The stop codon recognition was known as the first important step, while the contexts surrounding the stop codons were well known as a crucial determinant of the translation termination efficiency [28-31]. To elucidate the sequence patterns that could affect the efficiency of translation termination, the bases around the stop codons (−6 to +12) in the catfish full-length transcripts were examined. As illustrated in Figure 9, the bases around stop codon were biased. In particular, the −2 positions were biased toward A/U and the +4 positions were preferred for purines (A/G). The results we found in catfish were in agreement with previous studies in other eukaryotes, such as human, mouse, fruit fly and worm [32,33]. Numerous studies indicated that the nucleotide immediately following the stop codon (defined as +4) was crucial for termination and was biased toward purines [30,32]. Translation termination was also influenced by the sequence elements immediately upstream of the stop codons (−2 and −1 bases) as indicated in many studies (e.g., [34]), and the −2 positions were biased toward A and/or U in several eukaryotes previously examined [33]. Of the three stop codons, the usage frequency of the UGA is much higher than that of UAA and UAG in catfish (UGA 48.5%, UAA 32.5% and UAG 19%), which was consistent with general frequency of use of stop codons in eukaryotes [33].

To our best knowledge, there were no systematic studies on the sequence contexts surrounding the stop codons in fish species. However, we reexamined our previously identified 1,087 full-length cDNAs generated from clone sequencing to reconfirm the results we provided here. As shown in Additional file 6, the contexts around stop codons were highly consistent with the present results in Figure 9. The frequency of usage of the three stop codons were 45.4%, 38.6% and 16% for UGA, UAA and UAG, respectively, also similar as frequencies found from this study. This also provided additional support for the proper identification of the ORFs in this study.

### Polyadenylation signal (PAS) in the catfish transcripts

The polyadenylation signal (PAS) plays an important role in polyadenylation by determining the site for addition of a polyA tail to pre-mRNA. The PAS in mammals has been widely investigated and has been identified as the canonical hexamer AAUAAA which is located 10–30 nucleotide upstream of the cleavage site [35,36]. Although the AAUAAA signal is considered to be highly conserved, studies have shown that different variants of the PAS are certainly present in the 3’ ends of transcripts, and that the frequency distribution of the most common PAS versus alternate signals is species- and tissue-dependent [36,37]. The high diversity of gene expression of limited number of genes can be due to the 3’ end polyadenylation of pre-mRNA [38]. The choice of which element is used as PAS might play important roles in gene regulation, possibly by including or excluding down-stream regulatory motifs situated between such alternate PAS, as well as mRNA stability [35-37].

For the identification of PAS in catfish transcripts, 2,047 transcripts with at least seven adenosines at the end were selected for this analysis. Different variants of the PAS were observed in catfish. The most common observed immediate upstream of the polyA tail (within 35 bp) was the canonical AAUAAA (1,264 transcripts, 61%). The second most common variant was AUUAAA, present in 435 transcripts and accounting for 21% (Table 7), similar results as obtained in previous work [15]. These findings are in agreement with PAS motif frequency distribution in other species. Previous studies in mammals for the incidences of AAUAAA and AUUAAA polyadenylation signals in 3’ ends of cDNAs from EST sources have revealed that AAUAAA and AUUAAA are the two most common polyadenylation signals with the percentages of 50–60% and 10-15%, respectively [37]. In order to find the most frequently occurring hexamers in the remaining catfish transcripts, the TEIRESIAS algorithm [39] was used. The results showed that no dominant hexamer present in the remaining transcripts, rather several additional hexamers that share sequence similarity with canonical PAS sequence were identified. Table 7 lists the twelve most frequently occurring hexamers (single-base variants of AAUAAA) present in 10 or more transcripts. Of these putative PASs, five hexamers have been reported as PAS variants in salmon or catfish transcripts previously [15,25], eight of them have been identified in human genes based on EST resources [35].

**Table 7:** Identification and analysis of the catfish polyadenylation signal (PAS)

**Hexamer**	**Number of transcripts**	**Found in catfish^1^**	**Found in salmon^2^**	**Found in human^3^**
AAUAAA	1,264	+	+	+
AUUAAA	435	+	+	+
AAAAAA	47	+		
UAUAAA	25	+	+	+
AAGAAA	23		+	+
AAUATA	20	+	+	+
CAUAAA	19			+
AACAAA	18			
AAUACA	18			+
AAUGAA	16			+
AGUAAA	15	+		+
AAUAAU	10			
ACUAAA	10			+
AAUUAA	10			

A subset of the full-length transcripts with polyA tails generated from sequencing were selected for analysis. Most common PAS alternatives (hexamers with single-base variant of AAUAAA) were identified within 35 nucleotides upstream of the polyA tail from full-length transcripts. ^1^Alternative PAS identified in Chen et al. (2010) [15]; ^2^ Alternative PAS identified in Andreassen et al. (2009) [25]; and ^3^Alternative PAS identified in Macdonald et al. (2002) [37].

### Regulatory motifs in UTR regions of catfish full-length transcripts

Regulatory motifs are short nucleotide sequences that regulate gene expression. Most of the regulatory motifs are thought to be embedded in the non-coding part of the genomes. Among non-coding regions, the 5' and 3' UTRs of eukaryotic mRNAs have often been experimentally demonstrated to contain sequence elements crucial for many aspects of gene regulation and expression [40]. The large set of full-length transcripts generated in this study provided the opportunity for the identification of conserved regulatory motifs in the UTR regions of catfish transcripts. All 5’ and 3’ UTRs from catfish full-length transcripts were searched against UTRsite collection by using the pattern match program UTRscan. The UTRsite is a collection of functional sequence patterns located in 5' or 3' UTR sequences whose function and structure have been experimentally determined and published [40].

As summarized in Table 8, a total of 21 regulatory motifs were identified from catfish UTRs. For instance, the analysis of the 5’ and 3’ UTRs revealed transcripts with a motif that matched the iron responsive element (IRE). IRE, a particular hairpin structure located in the 5' UTR or 3' UTR of various mRNAs coding for proteins involved in cellular iron metabolism, is recognized by trans-acting proteins known as iron regulatory proteins that regulate mRNA translation rate and stability [41]. This evolutionary conserved motif was known to be present in the ferritin genes of vertebrates [42]. Our observation that presence of an IRE located in the 5’ UTR of catfish ferritin supported the idea that the motif identified was a true functional IRE. The analysis of the 3’ UTRs of the full-length cDNAs also revealed transcripts with motifs that matched Selenocysteine Insertion Sequences (SECIS). The SECIS element is a specific 60 bp stem-loop structure located in 3’ UTRs of mRNAs, and required for decoding UGA selenocysteine instead of termination of translation [43]. Catfish transcripts with matches to the SECIS element encoded selenium-related genes, such as glutathione peroxidase [44], defender against cell death protein [45], selenoprotein and Glutaredoxin [46], also suggesting the correct identification of this functional element.

**Table 8:** Identification of conserved regulatory motifs from catfish untranslated regions (UTRs)

**Regulatory motifs**	**Standard name**	**Location (UTR)**	**Number of UTRs**
IRE	Iron Responsive Element	5' and 3’	13
IRES	Internal Ribosome Entry Site	5'	2,491
SXL_BS	SXL binding site	5' and 3’	1,469
TOP	Terminal Oligopyrimidine Tract	5'	265
UNR-bs	UNR binding site	5' and 3’	907
uORF	Upstream Open Reading Frame	5'	5,492
15-LOX-DICE	15-Lipoxygenase Differentiation Control Element	3'	2
ADH_DRE	Alcohol dehydrogenase 3'UTR downregulation control element	3'	91
ARE2	AU-rich class-2 Element	3'	82
BRD-BOX	Brd-Box	3'	578
BRE	Bruno 3'UTR responsive element	3'	28
CPE	Cytoplasmic polyadenylation element	3'	180
G3A	Elastin G3A 3'UTR stability motif	3'	2
GLUT1	Glusose transporter type-1 3'UTR cis-acting element	3'	2
GY-BOX	GY-Box	3'	232
INS_SCE	Insulin 3'UTR stability element	3'	1
K-BOX	K-Box	3'	919
MBE	Musashi binding element	3'	3,546
SECIS1	Selenocysteine Insertion Sequence - type 1	3'	42
SECIS2	Selenocysteine Insertion Sequence - type 2	3'	36
TGE	TGE translational regulation element	3'	5

Catfish conserved regulatory motifs were identified by comparison with experimentally validated regulatory motifs deposited in UTRsite database using pattern search program UTRscan. Number of UTRs denotes the number of catfish UTRs from which the corresponding regulatory motifs were identified.

## Discussion

The transcriptome sequencing enables various structural and functional genomic studies of an organism. Although a lot of Sanger-based EST sequencing projects had been carried out for comprehensive characterization of transcriptomes, expressed sequence data are still limited resources, specifically in non-model species. The next-generation sequencing technologies provide a low cost, labor-saving and rapid means for transcriptome sequencing and characterization. However, the *de novo* assembly of short reads without a known reference is still difficult [47]. High throughput 454 sequencing which generates longer reads has been widely used in many transcriptome sequencing studies in non-model species previously [48,49]. Recently, more and more studies have shown the feasibility of transcriptome assembly by using Illumina short reads [12], especially with the combination of paired-end reads sequencing technology to facilitate the assembly. However, the differential gene expression results in variable coverage in transcriptome sequencing, the choice of a single *k*-mer value usually used in genome assembly cannot generate an assembly with emphasis on both transcript diversity and contiguity. Performing multiple assemblies with various *k*-mer lengths and to retain the best part of each one to form the final assembly has been shown effective for *de novo* transcriptome assembly [50]. Furthermore, as each assembler utilizes different approaches to deal with sequencing errors and paired-end information, the assemblers may differ in their abilities to capture different portions of the transcriptome with accuracy. It is reasonable that merging assemblies from multiple assemblers might yield a combined assembly with higher accuracy. More comprehensive transcripts would be obtained with combinatory use of several *de novo* assemblers.

In this study, we reported an efficient assembly and annotation of the catfish transcriptome by applying several combined strategies. Firstly, we took advantage of a doubled haploid channel catfish to reduce the complexity of transcriptome. Most importantly, the doubled haploid allows biologically meaningful assembly of transcripts without artificially creating “haplotypes” that do not exist in nature. The sequence assembly was facilitated since there are no allelic variations. Various tissues were collected with the aim to cover a comprehensive transcriptome. The paired-end reads were generated to resolve the assembly problem caused by repetitive regions. Secondly, we generated a final assembly with a combinatory use of three different widely used *de novo* assemblers. Multiple *k*-mer method was also used to enable the assembly sensitivity and contiguity. A higher N50 length and average length are considered as a benchmark for better assembly on contiguity. Our results showed that N50 length and average length of contigs varied greatly as a function of *k*-mer length, and also varied greatly between different assemblers. To get the optimum results, the validation of different assembly programs was conducted by comparing sequence similarity with closely related species. The ABySS generated contigs with higher N50 length and average length indicating its strength in generating assembly with better contiguity, while the Velvet generated sequences with more number and percentage of contigs showed significant similarity with zebrafish proteins. The CLC Genomics Workbench performed as intermediate between ABySS and Velvet according to both contiguity and sensitivity. The final assembly obtained by merging all these three sets of assembly provided a more comprehensive and accurate assembly.

**Figure 10:**
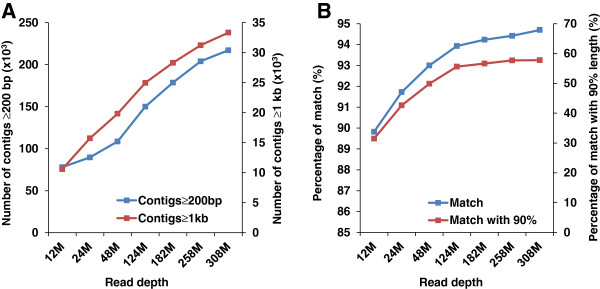
**Evaluation of sequencing depth for the assembly of the catfish transcriptome assembly. **(**A**) Assemblies were evaluated based on the number of assembled contigs with length ≥ 200 bp and 1 kb. The X-axis represents assemblies with various sequencing depths generated by CLC Genomics Workbench, left Y-axis represents the number of contigs with length ≥ 200 bp in thousand, and right Y-axis represents the number of contigs with length ≥ 1 kb in thousand. (**B**) Assemblies were evaluated based on the number of zebrafish proteins that were identified in the assembled catfish contigs. The X-axis represents assemblies with various sequencing depths assembled by CLC Genomics Workbench, left Y-axis represents the percentage of zebrafish proteins that can be detected in catfish and right Y-axis represents the percentage of zebrafish proteins that can be detected in catfish with match length ≥ 90%.

We believe that the sequencing depth was sufficient to cover the vast majority of transcripts. To assess the depth of sequencing obtained for transcriptome assembly in this work, three lanes of sequencing was conducted with one of which being sequenced for around one fourth yield of a whole lane (Table 1) and the sequence datasets were resampled into several sub-datasets with various read depths (Additional file 7). The *de novo* assemblies of these sub-datasets were generated using CLC Genomics Workbench to determine the effects of read depths on the transcriptome assembly. The summary of assembly statistics was given in Addition file 6. The number of contigs with minimum length of 200 bp and 1 kb were collected as two benchmarks for the assembly sensitivity and continuity. As shown in Figure 10A, with the increase of sequencing depth, the number of contigs with minimum length of 200 bp increased. A significant increase of transcriptome coverage (assembly sensitivity) was observed from 48M to 124M. Relatively slight increase was observed when the number of reads increased from 258M to 308M, but nonetheless the number of assembled contigs with sizes greater than 200 bp or 1 kb continues to grow as the sequence depth increased. This was somewhat outside of our expectation, perhaps due to the segmented assemblies. We therefore decided to determine if the percentage of gene hits continue to increase with the increase of sequencing depth.

The transcriptome coverage and completeness achieved by these sub-assemblies were then evaluated by matching with annotated known genes. Due to the limited number of catfish genes in the public database, we used the zebrafish RefSeq protein sequences in the NCBI database to conduct this evaluation. There are a total of 27,239 zebrafish annotated protein sequences available which were searched against the assembled catfish contigs for homologous match using TBLASTN. The numbers of detected zebrafish proteins at different levels of sequencing depth are presented in Additional file 8. There were 25,795 zebrafish proteins observed in the catfish RNA-Seq assembly accounting for 94.7% of all annotated proteins in the database. As shown in Figure 10B, the number of observed genes increased almost linearly with lower sequencing depth. However, the number of observed genes started to plateau when the sequencing depth reached 124 million reads. From 124 million reads to 308 million reads, the percent of gene hits increased from 94% to 95%; while the percentage of gene hits with greater than 90% length homology stayed essentially unchanged, suggesting that the sequencing depth was sufficient to provide a good coverage of the transcriptome.

The analysis of sequence conservation comparing with other model fish species helps in transfer of knowledge from model species to catfish for both structural and functional genomic studies. A large number of catfish transcripts showed significant similarity with model fish at protein level as expected, suggesting that their function might also be conserved. Interestingly, a large number of the transcripts did not show significant homology with any other reference sequences, which may be novel and transcribed from catfish-specific genes. The study of these genes will be very important to dissect the species-specific cellular process, ‘catfish-specific’ gene duplication and divergence and study evolutionary processes of speciation and adaptation.

The doubled haploid fish used in this work provided an opportunity to evaluate genome-scale gene duplication in catfish. There were a total of 2,659 unique genes detected as putative duplicated genes. Ultimately, duplicated genes will have different genome coordinates as to their locations. However, the catfish genome sequencing is still in progress. Nonetheless, the evaluation based on the catfish preliminary genome assembly (unpublished) supported that the majority of these genes had duplicate copies. Clearly, the use of doubled haploid catfish was not only important for the transcript assembly, but also important for the initial identification of putative gene duplications in catfish. Additional analysis and validation are needed to demonstrate that the putative duplicated genes are indeed duplicated in the future.

## Conclusions

In conclusion, we have demonstrated the use of short-reads sequence data to efficiently and comprehensively characterize a draft transcriptome of an organism without sequenced genome. The strategy of *de novo* assembly described here can be potentially used for other species. The advantages offered by the use of a homozygote are applicable to most teleost species where doubled haploid can be made. Our study contributed a significant non-redundant set of 370,798 transcripts including 14,240 full-length transcripts in catfish. The detailed analyses of these sequences has provided several important features of catfish transcriptome such as length distribution, sequence patterns around translation initiation and termination codons, conserved regulatory motifs, conserved genes across fishes, and functional annotation. It is anticipated that the results from this study will contribute significantly towards assembly and annotation of the catfish genome. Such resources will likely be important for structural and functional genomics studies in other teleosts and related species as well.

## Methods

### Sample and RNA isolation

To better characterize the catfish transcriptome and improve discovery of variations derived from duplicated genes, a haploid transcriptome is needed. Doubled haploid channel catfish, that have two identical copies of each chromosome, were established using gynogenesis [16]. Tissues were collected from a single doubled haploid female channel catfish adult for this study. The fish were euthanized with tricaine methanesulfonate (MS 222) at 300 mg/l before tissue collection. Samples of 19 tissues including head kidney, fin, pancreas, spleen, gill, brain, trunk kidney, adipose, liver, stomach, gall bladder, ovary, intestine, thymus, skin, eye, swim bladder, muscle, and heart were collected. Tissues were flash-frozen in liquid nitrogen and shipped on dry ice then stored at −80°C until RNA extraction. Tissue samples were ground to a fine powder with mortar and pestle in the presence of liquid nitrogen and thoroughly mixed. A fraction of the tissue samples was used for RNA isolation. Total RNA was isolated using the RNeasy plus Mini Kit (Qiagen, USA) followed by DNase I (Invitrogen, USA) treatment according to the manufacturer's protocol as in previous study [5]. Equal amount of total RNA from each tissue was combined and sent out for commercial sequencing.

### Illumina sequencing

Sequencing was conducted commercially in HudsonAlpha Genomic Services Lab (Huntsville, AL, USA) similarly as in previous study [5]. Briefly, 100 ng of total RNA was used to prepare amplified cDNA using Ovation RNA-Seq, a commercially available kit optimized for RNA sequencing (NuGEN Technologies, San Carlos, CA). The produced double-stranded cDNA was subsequently used as the input to the Illumina library preparation protocol starting with the standard end-repair step. The end-repaired DNA with a single ‘A’-base overhang is ligated to the adaptors in a standard ligation reaction using T4 DNA ligase and 2 μM-4 μM final adaptor concentration, depending on the DNA yield following purification after the addition of the ‘A’-base. Following ligation, the samples were purified and subjected to size selection via gel electrophoresis to isolate 350 bp fragments for ligation-mediated PCR (LM-PCR). Twelve cycles of LM-PCR were used to amplify the ligated material in preparation for cluster generation. The prepared cDNA library was sequenced for 100-bp paired-end reads on three flow cell lanes using the Hiseq 2000 platform, but one of the three lanes was partitioned for including three other samples, generating less number of sequences as in other two lanes for catfish. The image analysis, base calling and quality score calibration were processed using the Illumina Pipeline Software v1.4.1 according to the manufacturer's instructions. Reads were exported in FASTQ format and has been deposited at the NCBI Sequence Read Archive (SRA) under accession number SRA047025.

### Assembly of expressed short reads

The raw reads were cleaned by trimming of adaptor sequences, ambiguous nucleotides (‘N’ in the end of reads) and low quality sequences with average quality scores less than 20. Reads less than 15 bp after trim were also discarded, the remaining reads were used in subsequent assembly. In order to obtain a comprehensive and reliable assembly, three different assemblers including CLC Genomics Workbench (version 4.2; CLC bio, Aarhus, Denmark), ABySS (version 1.2.6) and Velvet (version 1.1.02) were used for *de novo* assembly. In brief, the CLC *de novo* assembly was performed with a choice of an optimized *k*-mer length based on the input data by default settings. In case of ABySS and Velvet assembly, multiple *k*-mer approach with every other *k*-mer values from 21 to 95 for ABySS and from 45 to 95 for Velvet were used so as to maximize assembly contiguity and sensitivity. Subsequently, the multiple *k*-mer assemblies from ABySS and Velvet were merged by running the first stage of the trans-ABySS analysis pipeline (version 1.2.0) [51], respectively. Afterwards, these three assemblies were combined to produce the final non-redundant assembly. As anticipated, some identical contigs were generated from more than one assemblies introducing duplicates. The CD-HIT-EST [52] was used to remove redundancy and retain the longest possible contigs. The short redundant contigs were removed, and the remaining contigs composed the final assembly of non-redundant transcripts.

### Functional annotation and identification of putative full-length transcripts

All the non-redundant transcripts from final assembly were searched against NCBI zebrafish RefSeq protein database and Uniprot/Swiss-Prot database using BLASTX with E-value ≤ 1e-10. The ORFs were predicted with the software orfPredictor [53] by using BLASTX as a guide for the prediction. The BLASTX-aided method detects ORFs by finding the starting methionine and stop codon in catfish transcripts relative to the same features in the most closely related species identified by BLASTX. In the cases where the catfish transcripts did not show high similarity to reference protein, ORF identified by finding the longest stretch of uninterrupted sequence between a start codon and a stop codon in both strand orientations. The completeness of ORFs in each transcript sequence was determined by the BLASTX alignment. We considered a full-length transcript to contain a complete CDS if the ORF revealed a start codon and stop codon in agreement with the match in the database. In the context of this work, the full-length transcript was defined as a consensus sequence containing the complete CDS and at least partial 5’ and 3’ UTR sequence. The start and stop codons of CDSs were used to define the boundary between the CDSs and the 5' and 3' UTRs. If a significant match did not contain a start codon in the 5' or a stop codon in the 3' end of the coding sequence and the pairwise alignment indicated that the transcript lacked some 5' or 3' coding sequence, it was considered to be a transcript with a partial coding sequence.

### Detection of catfish putative gene duplicates

Since the doubled haploid channel catfish were used, there should be no allelic variations, and the gene-derived transcripts showing signs of “SNPs” would be assembled from duplicate gene copies. In order to detect the PSVs or MSVs derived from the duplicated genes, the catfish transcripts that had unique protein hits were used as reference, and all the short reads were mapped with the similarity of 99%. The “SNPs” (actually the PSVs or MSVs) were detected as SNP detection in previous work [5]. Briefly, at least four short reads were required for “SNP” detection at each position, the minimum number of variant alleles was required as at least two, and minor allele frequency was required as at least 10%. Putative catfish duplicated genes were assessed by aligning the transcripts with the preliminary catfish whole genome assembly (unpublished data) using BLASTN with the E-value cutoff of 1e-10 and minimum alignment length of 100 bp.

### Analysis of UTRs of full-length transcripts

The catfish Kozak consensus sequences were examined in the 5’ UTR analysis. Eight-base sequences spanning from position −4 to position +4 of transcripts were selected. The extracted sequences were used as input into WebLogo [23] to assess the common Kozak consensus sequence in catfish transcripts.

For 3’ UTR analysis, the TEIRESIAS-based pattern discovery tool [39] was used to search the most frequently occurring motifs. A search for putative polyadenylation signals (PAS) in full-length transcripts was performed using 35 bp sequence immediate upstream of the polyA tail as input. Pattern discovery tool conditions in the program were: “exact discovery”, L=6 and W=6. To elucidate the sequence patterns that could affect the efficiency of translation termination, the bases around the stop codons (−6 to +12) in the catfish full-length transcripts were extracted and illustrated using WebLogo [23].

To identify evolutionarily conserved regulatory motifs in catfish transcripts, we searched the UTR database collection (UTRdb) [40] using the 5’ and 3’ UTRs as queries with the pattern match program UTRscan.

## Competing interests

The authors declare that they have no competing interests.

## Authors’ contributions

SL conducted the major part of the research including preparation of the samples, bioinformatic analysis and manuscript preparation. YZ, ZZ, FS, JL, JZ, YJ, HZ, XW, RK, and HK were involved in one or more processes of RNA extraction or bioinformatic analysis. GW generated the doubled haploid fish and prepared tissue samples for this work. LK provided assistance for the tissues preparation. EP provided assistance for data analysis and manuscript preparation. ZL supervised the entire study and provided assistance for data analysis and manuscript preparation. All authors read and approved the final manuscript.

## Supplementary Material

Additional file 1 **Table BLASTX annotation of three assemblies from various *de novo *assemblers.** Three assemblies, generated from CLC Genomics Workbench, ABySS, and Velvet respectively, were blasted against zebrafish RefSeq protein and Uniprot/Swiss-Prot databases, with the E-value cutoff of 1e-10. Click here for file

Additional file 2 **Figure ORF length distribution for contigs without significant protein hits from public protein database.** X-axis represents the predicted ORF length in amino acids, and Y-axis is the number of catfish contigs. Click here for file

Additional file 3 **Table Results of conserved domain finding for contigs without protein hits by homology search.** The predicted ORFs from the contigs without significant BLASTX hits were searched against the NCBI Conserved Domain database using the CD-search tool with the default settings. Click here for file

Additional file 4 **Figure Schematic presentation of principles for detection of putative catfish gene duplicates.** The reconstructed transcripts from protein-coding genes that show signs of “SNPs” (PSVs/MSVs) can be assembled by short reads from duplicated genes. Click here for file

Additional file 5 **Table Detection of putative gene duplicates in catfish and comparison with the preliminary catfish genome assembly.** The catfish gene duplicates were detected as the ones showing signs of PSVs/MSVs, and the evaluation of these duplicated genes was achieved by comparing with the preliminary catfish genome assembly. Click here for file

Additional file 6 **Figure Sequence contexts around stop codon of previously identified full-length cDNAs.** The sequence contexts surrounding the stop codon of 1,087 previously identified full-length cDNAs were illustrated using WebLogo. Click here for file

Additional file 7 **Table Summary of sub-assemblies statistics for various sequencing read depths.** The sequencing data were sub-sampled into several different sequencing read depths including 12 million, 24 million, 48 million, 124 million, 182 million, 258 million, and 308 million reads. These sub-datasets were assembled using CLC Genomics Workbench to evaluate the effect of sequencing read depth on catfish transcriptome assembly. Click here for file

Additional file 8 **Table Summary of sub-assemblies compared with zebrafish proteins using TBLASTN.** The sub-assemblies assembled from reads of several different sequencing read depths were assessed for the number of genes covered by comparing with NCBI zebrafish RefSeq proteins with the E-value cutoff of 1e-10. Click here for file
