# Supplementary material for: Efficient assembly and annotation of the transcriptome of catfish by RNA-Seq analysis of a doubled haploid homozygote
Source: BMC Genomics. 2012 Nov 5;13:595. doi: 10.1186/1471-2164-13-595 (PMC3582483; doi:10.1186/1471-2164-13-595)
Supplement: Additional file 1 — Table BLASTX annotation of three assemblies from various de novo assemblers. Three assemblies, generated from CLC Genomics Workbench, ABySS, and Velvet respectively, were blasted against zebrafish RefSeq protein and Uniprot/Swiss-Prot databases, with the E-value cutoff of 1e-10. [file 1471-2164-13-595-S1.pdf]

| Assembly | zebrafish Refseq protein No. of contigs with hits | zebrafish Refseq protein No. of unique protein hits | Uniprot protein No. of contigs with hits | Uniprot protein No. of unique protein hits | Total No. of contigs with hits | Total No. of unique protein hits |
|---|---|---|---|---|---|---|
| Velvet | 64,343 | 19,011 | 56,235 | 15,714 | 68,043 | 22,710 |
| Abyss | 66,666 | 18,174 | 59,761 | 14,544 | 68,805 | 21,126 |
| CLC | 35,395 | 18,176 | 33,629 | 16,410 | 39,898 | 22,047 |
