# Supplementary figures and images for: Efficient assembly and annotation of the transcriptome of catfish by RNA-Seq analysis of a doubled haploid homozygote
Source: BMC Genomics. 2012 Nov 5;13:595. doi: 10.1186/1471-2164-13-595 (PMC3582483; doi:10.1186/1471-2164-13-595)

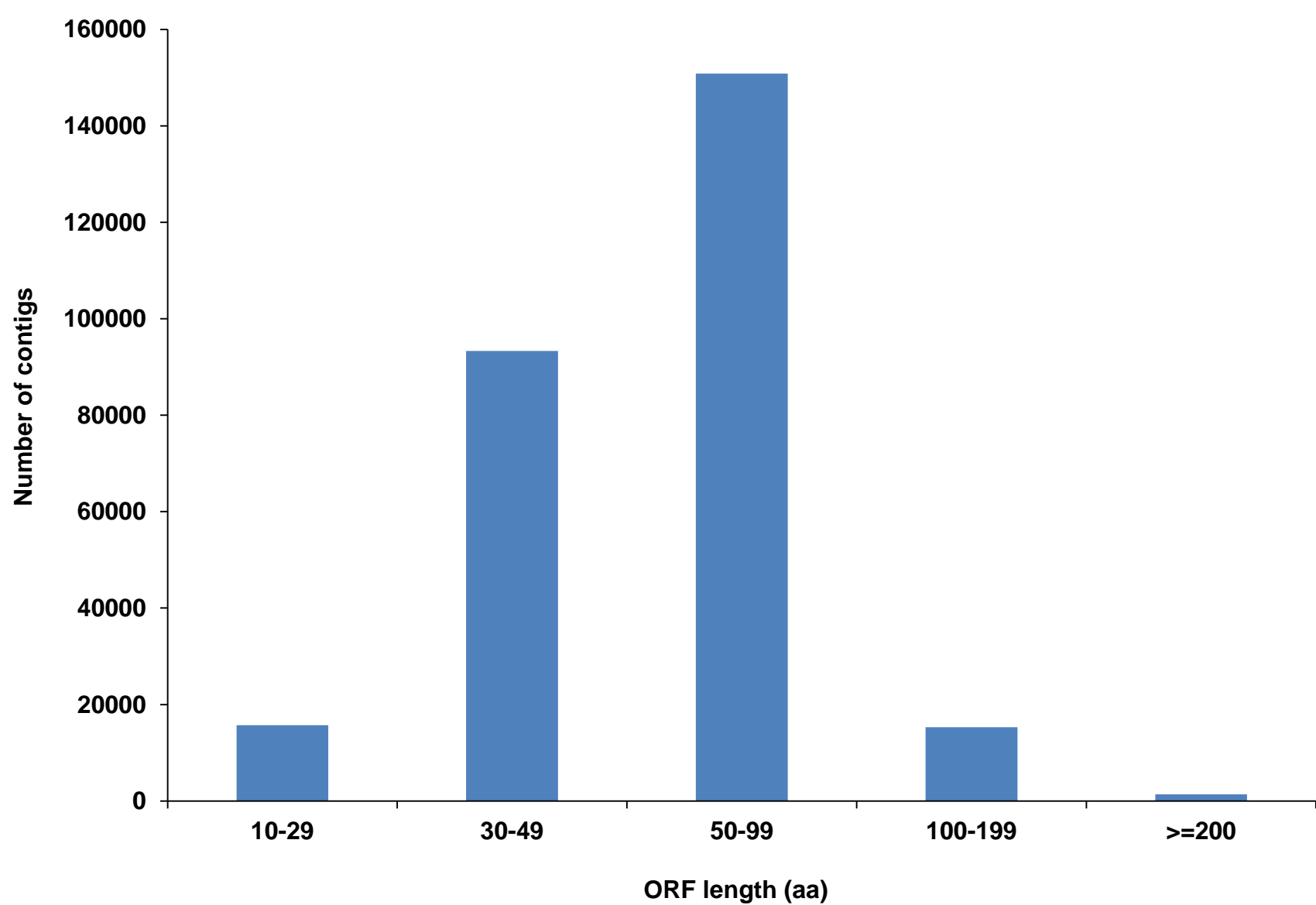

Supplement: Additional file 2 — Figure ORF length distribution for contigs without significant protein hits from public protein database. X-axis represents the predicted ORF length in amino acids, and Y-axis is the number of catfish contigs. [file 1471-2164-13-595-S2.pdf]

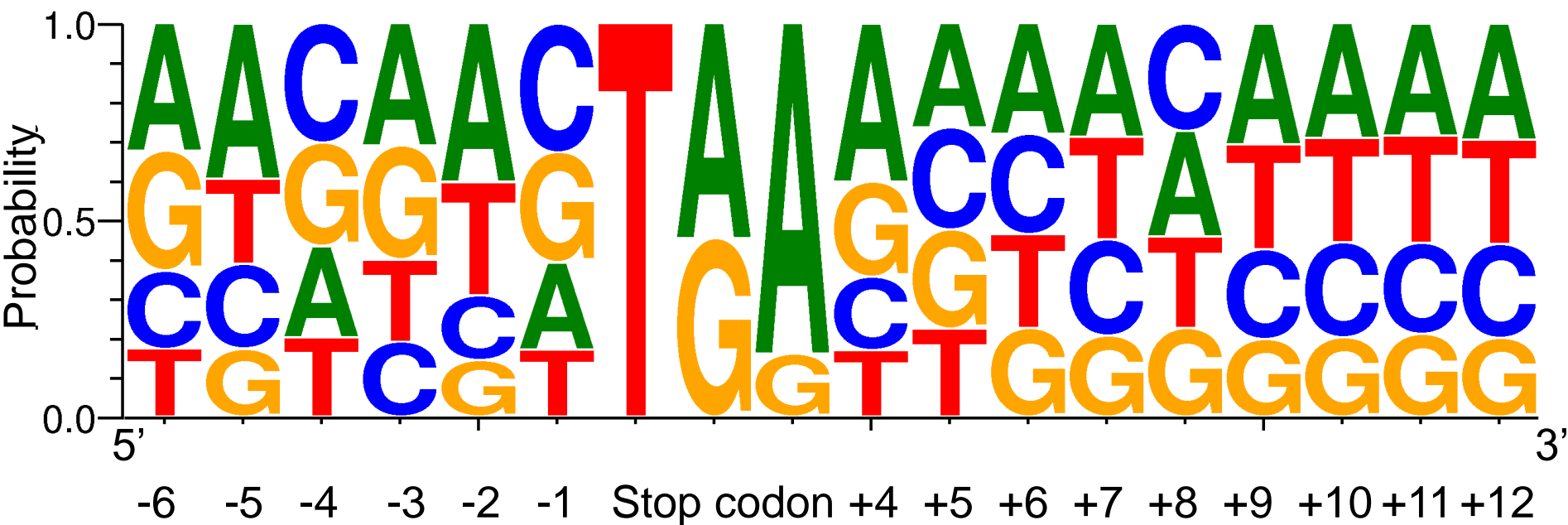

Supplement: Additional file 6 — Figure Sequence contexts around stop codon of previously identified full-length cDNAs. The sequence contexts surrounding the stop codon of 1,087 previously identified full-length cDNAs were illustrated using WebLogo. [file 1471-2164-13-595-S6.pdf]
