# Supplementary material for: Efficient assembly and annotation of the transcriptome of catfish by RNA-Seq analysis of a doubled haploid homozygote
Source: BMC Genomics. 2012 Nov 5;13:595. doi: 10.1186/1471-2164-13-595 (PMC3582483; doi:10.1186/1471-2164-13-595)
Supplement: Additional file 3 — Table Results of conserved domain finding for contigs without protein hits by homology search. The predicted ORFs from the contigs without significant BLASTX hits were searched against the NCBI Conserved Domain database using the CD-search tool with the default settings. [file 1471-2164-13-595-S3.pdf]

	Number
**Catfish contigs without blast hits (1e-10)**	276,322
**Contigs had ORFs detected (Minimum length of 30 amino acids)**	260,793
**Contigs had ORFs detected (Minimum length of 100 amino acids)**	16,688
**Contigs had Conserved Domains identified by CD-Search in NCBI**	**4,984**
