# Supplementary material for: Efficient assembly and annotation of the transcriptome of catfish by RNA-Seq analysis of a doubled haploid homozygote
Source: BMC Genomics. 2012 Nov 5;13:595. doi: 10.1186/1471-2164-13-595 (PMC3582483; doi:10.1186/1471-2164-13-595)
Supplement: Additional file 4 — Figure Schematic presentation of principles for detection of putative catfish gene duplicates. The reconstructed transcripts from protein-coding genes that show signs of “SNPs” (PSVs/MSVs) can be assembled by short reads from duplicated genes. [file 1471-2164-13-595-S4.pdf]

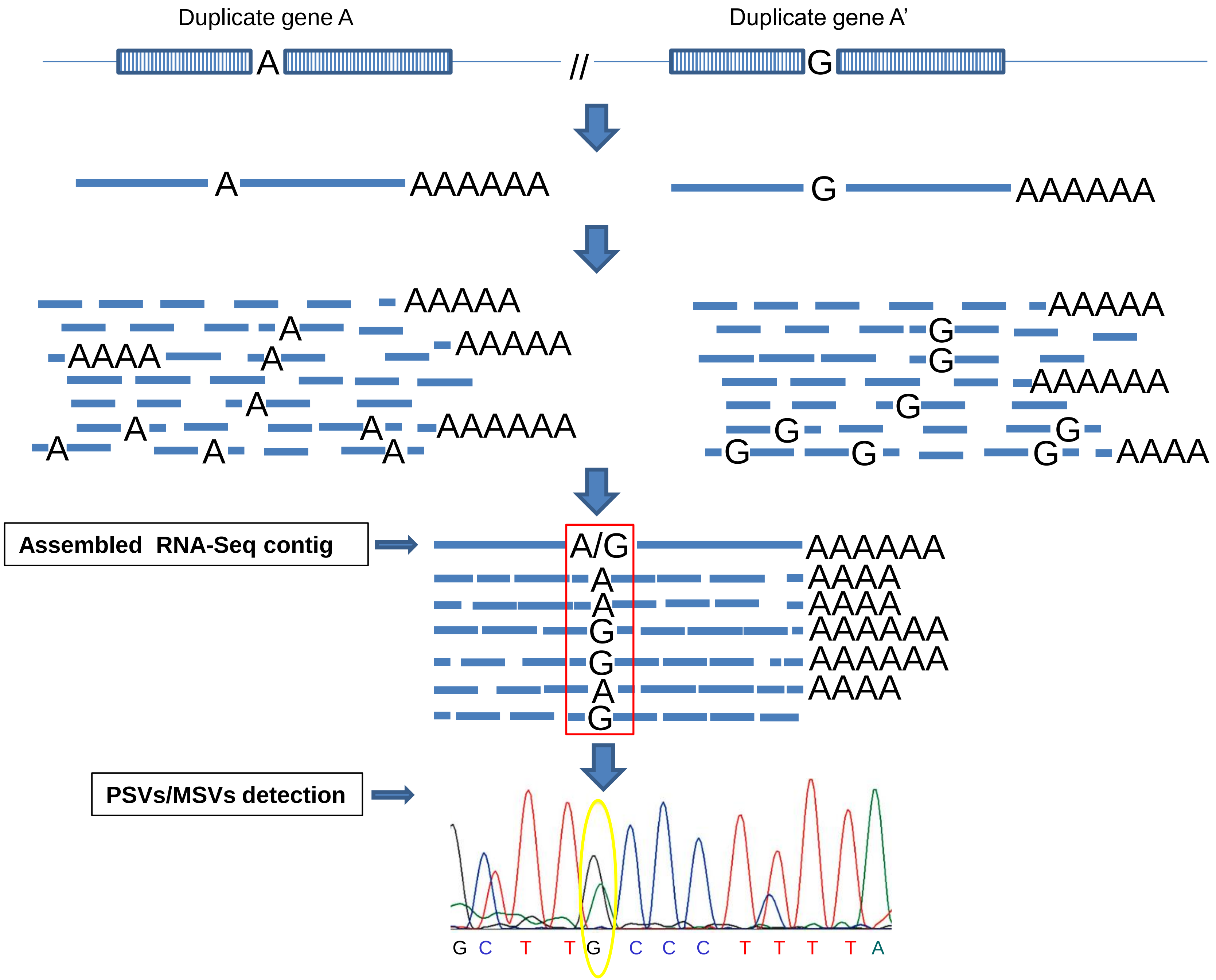

Duplicate gene A
Duplicate gene A'
A
G
//
AAAAAA
Assembled RNA-Seq contig
A/G
PSVs/MSVs detection
G C T T G C C C T T T T A
