# Supplementary material for: Efficient assembly and annotation of the transcriptome of catfish by RNA-Seq analysis of a doubled haploid homozygote
Source: BMC Genomics. 2012 Nov 5;13:595. doi: 10.1186/1471-2164-13-595 (PMC3582483; doi:10.1186/1471-2164-13-595)
Supplement: Additional file 5 — Table Detection of putative gene duplicates in catfish and comparison with the preliminary catfish genome assembly. The catfish gene duplicates were detected as the ones showing signs of PSVs/MSVs, and the evaluation of these duplicated genes was achieved by comparing with the preliminary catfish genome assembly. [file 1471-2164-13-595-S5.pdf]

	Number
Catfish transcripts with unique protein hits	25,144
Catfish unique genes	23,139
PSVs/MSVs detected from catfish putative gene duplicates	4,878
Transcripts with detection of PSVs/MSVs	2,692
Total unique genes with detection of PSVs/MSVs	2,659
Unique genes with detection of 1 PSV/MSV	1,789
Unique genes with detection of 2 PSVs/MSVs	410
Unique genes with detection of 3 PSVs/MSVs	194
Unique genes with detection of 4 PSVs/MSVs	97
Unique genes with detection of 5 PSVs/MSVs	56
Unique genes with detection of ⩾6 PSVs/MSVs	113
Putative catfish gene duplicates had ⩾2 hits on genome contigs	2,446
Putative catfish gene duplicates had only one hits on genome contigs	196
Putative catfish gene duplicates had no hits on genome contigs	17
