# Supplementary material for: Efficient assembly and annotation of the transcriptome of catfish by RNA-Seq analysis of a doubled haploid homozygote
Source: BMC Genomics. 2012 Nov 5;13:595. doi: 10.1186/1471-2164-13-595 (PMC3582483; doi:10.1186/1471-2164-13-595)
Supplement: Additional file 7 — Table Summary of sub-assemblies statistics for various sequencing read depths. The sequencing data were sub-sampled into several different sequencing read depths including 12 million, 24 million, 48 million, 124 million, 182 million, 258 million, and 308 million reads. These sub-datasets were assembled using CLC Genomics Workbench to evaluate the effect of sequencing read depth on catfish transcriptome assembly. [file 1471-2164-13-595-S7.pdf]

Sub-assembly	Number of catfish contigs matched with zebrafish reference proteins	%	Number of catfish contigs matched to 90% length of zebrafish reference proteins	%
12M	24,466	89.8	8,568	31.5
24M	24,984	91.7	11,609	42.6
48M	25,331	93.0	13,575	49.8
124M	25,586	93.9	15,146	55.6
182M	25,668	94.2	15,424	56.6
258M	25,719	94.4	15,712	57.7
308M	25,795	94.7	15,733	57.8
