# Supplementary material for: Efficient assembly and annotation of the transcriptome of catfish by RNA-Seq analysis of a doubled haploid homozygote
Source: BMC Genomics. 2012 Nov 5;13:595. doi: 10.1186/1471-2164-13-595 (PMC3582483; doi:10.1186/1471-2164-13-595)
Supplement: Additional file 8 — Table Summary of sub-assemblies compared with zebrafish proteins using TBLASTN. The sub-assemblies assembled from reads of several different sequencing read depths were assessed for the number of genes covered by comparing with NCBI zebrafish RefSeq proteins with the E-value cutoff of 1e-10. [file 1471-2164-13-595-S8.pdf]

Sub-datasets	Number of reads	Number of contigs with length ≥ 200bp	Number of contigs with length ≥ 1kb	Number of contigs with length ≥ N50	Average length (bp)	N50 (bp)	Maximum length	Total assembly size (Mb)
12M	12,188,270	78,006	10,585	14,123	579	799	14,420	45
24M	24,377,425	89,664	15,732	13,833	668	1,122	19,871	60
48M	48,743,295	108,653	19,836	14,936	704	1,331	24,878	77
124M	124,882,873	150,023	24,965	19,668	686	1,275	29,426	103
182M	182,670,036	178,608	28,312	23,656	669	1,193	26,392	120
258M	258,809,614	204,023	31,238	27,383	658	1,133	41,219	134
308M	307,552,909	217,153	33,332	29,550	657	1,120	38,652	143
